# Beyond Permeability Prediction: Machine Learning for Gas-Separation Membranes from Data Curation to Experimentally Validated Discovery

**DOI:** 10.3390/membranes16100340

**Published:** 2026-10-13

**Authors:** Zhengda Lin, Bingjie Yan, Xinhao Sun, Zhe Fu, Xingwei Du, Jun Zhang

**Affiliations:** 1 State Key Laboratory of Urban Water Resource and Environment, School of Environment, Harbin Institute of Technology, Harbin 150090, China; 2 Harbin Dan Shao Da Environmental Protection Technology Co., Ltd., Harbin 150090, China

**Keywords:** gas separation, membrane materials, machine learning, polymer informatics, metal–organic frameworks, mixed-gas validation, uncertainty quantification, inverse design

## Abstract

**Highlights:**

• A five-level evidence framework ranks ML findings by how strongly they are validated.
• A standardized record keeps membrane state, test conditions and provenance together.
• A validation ladder reveals inflated scores from similarity leakage and domain shift.
• An uncertainty-aware closed loop learns feasibility and fabrication failures.
• Decision-grade ML ranks candidates by membrane process utility, not prediction alone.

**Abstract:**

Machine learning (ML) is transforming gas-separation membrane research, yet high retrospective accuracy does not necessarily translate into experimentally viable or process-relevant materials. This review reframes the field around the evidence chain required to convert a membrane record into a defensible discovery decision. Across polymers, metal–organic and covalent organic frameworks, mixed-matrix membranes, carbon molecular sieves, and two-dimensional systems, we critically examine data curation, physics-aligned representation, multitask and multi-fidelity learning, uncertainty quantification, inverse design, and prospective validation. We introduce a five-level evidence framework spanning retrospective interpolation, domain-disjoint generalization, physics or simulation confirmation, prospective membrane validation, and deployment-relevant testing. We further propose a contextualized observation unit that binds chemical identity to membrane state, fabrication history, feed composition, operating conditions, time, protocol, and provenance, thereby exposing leakage and structural uncertainty hidden by conventional datasets. Our analysis shows that ML has demonstrably accelerated data completion, simulation, and candidate prioritization, whereas evidence for routinely discovering manufacturable, durable, mixed-gas-performing membranes remains limited and concentrated in polymer studies. To close this gap, we develop a constrained, uncertainty-aware closed-loop architecture that treats feasibility and fabrication failures as learnable outcomes, couples acquisition to multiscale process utility, and advances candidates through staged experimental fidelities. A minimum reporting standard and measurable research priorities are provided. Together, these contributions propose “decision-grade ML” as an emerging framework for evaluating machine learning in membrane science: models should know their applicability domain, preserve physical context, propose feasible experiments, quantify uncertainty under domain shift, and demonstrate value under the conditions of intended membrane use rather than merely reproduce familiar labels or idealized proxies.

## 1. Introduction: The Missing Link Between Prediction and Membrane Decisions

Gas separations impose a substantial energy and capital burden on chemical manufacturing, carbon capture, hydrogen purification, natural-gas upgrading and air separation [1]. This burden is central to large-scale carbon-capture deployment [2]. Membranes can reduce it because separation is driven by a chemical-potential gradient rather than by a phase change. Their practical value, however, depends on coupled material and process attributes rather than on permeability or selectivity in isolation [3,4]. System-level decarbonization analyses reinforce the need to connect material metrics to process performance [5].

A viable candidate must combine permeance and mixture selectivity with mechanical integrity, resistance to plasticization and physical aging, tolerance of water and trace contaminants, and manufacturability as a defect-controlled thin layer. Polymer membranes illustrate how transport is coupled to morphology and processing [6]. Porous-framework membranes add flexibility, defect and crystal-growth constraints [7,8], whereas composite and two-dimensional membranes introduce interfacial or scale-up limitations [9,10]. The familiar permeability–selectivity upper bounds remain indispensable maps of polymer performance [11,12]. They are not deployment criteria, particularly when mixture competition changes the apparent trade-off [13].

These variables are critical because a separation decision is made on the membrane as fabricated and operated, not on its idealized chemistry. Permeance sets the membrane area and compression duty; mixture selectivity determines product purity and recovery; resistance to plasticization and physical aging determines whether performance survives long-term mixed-gas operation; and manufacturability decides whether a laboratory coupon can become a defect-controlled thin layer at scale. The same chemical structure can therefore support or fail a separation depending on morphology, processing history and operating conditions: a humid post-combustion feed stresses water tolerance and thin-layer permeance, a high-pressure natural-gas feed stresses plasticization resistance and hydrocarbon competition, and hydrogen purification stresses temperature, pressure and competitive species. The permeability–selectivity upper bounds that summarize pure-gas polymer performance are accordingly necessary screening maps, but they are not deployment criteria: a candidate that ranks well in permeability and selectivity may still lose the decision to a lower-ranked material that is manufacturable, durable and competitive under the actual mixture and process conditions.

ML appears particularly attractive in this setting. Polymer and porous-crystal spaces are combinatorial, transport measurements are slow and heterogeneous, and molecular simulation remains too expensive for exhaustive screening. Early neural-network studies established that gas permeability could be learned from polymer descriptors [14,15]. Subsequent work used ML to complete sparse gas panels and to select polymers for prospective synthesis [16,17]. Large virtual screens and inverse-design studies then extended the search to millions of hypothetical polymers and porous frameworks [18,19]. Generative and explainable graph approaches have further linked candidate proposal to simulation or experiment [20,21].

These advances draw on polymer informatics, graph representation, data-efficient learning and inverse design [22,23]. Sustainable polymer design adds feasibility and lifecycle constraints to the search [24]. Recent reviews document the breadth of this rapidly developing field [25,26]. Membrane-design reviews likewise trace the progression from property prediction to AI-guided optimization [27,28]. Nevertheless, the literature still tends to equate predictive accuracy on a held-out subset with discovery capability.

That equivalence is unsafe. A model may interpolate successfully because related repeat units, near-duplicate frameworks or values extracted from the same source appear in both training and test sets. Redundancy-controlled evaluations show that removing such familiarity can materially change both model ranking and error [29]. A model may also rank candidates using pure-gas data measured on thick films even though the intended application involves a humid multicomponent feed and a submicrometer selective layer. Mixed-gas transport and physical aging make this transfer particularly uncertain [13,30].

A nominal chemical structure is likewise incomplete if molecular mass, crosslinking, casting solvent, thermal history, filler–polymer adhesion or defect density determine the realized membrane. Reviews that connect molecular screening to industrial performance therefore emphasize membrane formation and test context [31,32]. Even a chemically novel candidate can fail when its training labels combine incompatible pressure, temperature, aging time and permeability conventions. This is a provenance problem as well as a statistical one, and it conflicts with the traceability required by FAIR data practice [33]. The resulting failure is not merely dataset noise. It reflects a mismatch between the statistical observation and the physical decision.

Against this background, this review asks a decision-centered question: what evidence is required for an ML recommendation to survive membrane synthesis, realistic permeation testing and process evaluation? We focus on ML used to learn, predict, screen, design or optimize materials and material-enabled decisions for gas-separation membranes. The material scope includes polymers, MOFs, COFs, MMMs, CMS membranes and emerging atomically thin or two-dimensional systems. Process models are considered when they translate membrane properties from an ML or molecular-screening layer into a separation decision. General membrane-ML and polymer-informatics studies are used when they clarify representation, evaluation or uncertainty, whereas water treatment, pervaporation, adsorption-only systems and unrelated energy applications are not treated as evidence of gas-separation performance.

We performed a literature search in Web of Science, Scopus, PubMed and Google Scholar to identify the studies covered in this review, using combinations of keywords including “machine learning”, “artificial intelligence”, “gas separation”, “membrane”, “permeability”, “polymer informatics”, “metal–organic framework”, “covalent organic framework”, “mixed-matrix membrane”, “carbon molecular sieve”, “two-dimensional membrane”, “screening”, “inverse design” and “active learning”. The search covered peer-reviewed publications from 2000 up to June 2026 and was supplemented by citation tracking and manual screening of the reference lists of recent reviews and key primary studies. Articles were included when they (i) applied machine learning to learn, predict, screen, design or optimize materials for gas-separation membranes; (ii) provided methodological contributions relevant to representation, evaluation, uncertainty quantification or data curation for membrane machine learning; or (iii) reported membrane-relevant process or simulation studies that translate material properties into separation decisions. Studies on water treatment, pervaporation, adsorption-only systems and unrelated energy applications were excluded as evidence of gas-separation performance, consistent with the scope defined above. A small number of authoritative reports and books were consulted for domain context and are cited where standard concepts are defined.

**Figure 1:**
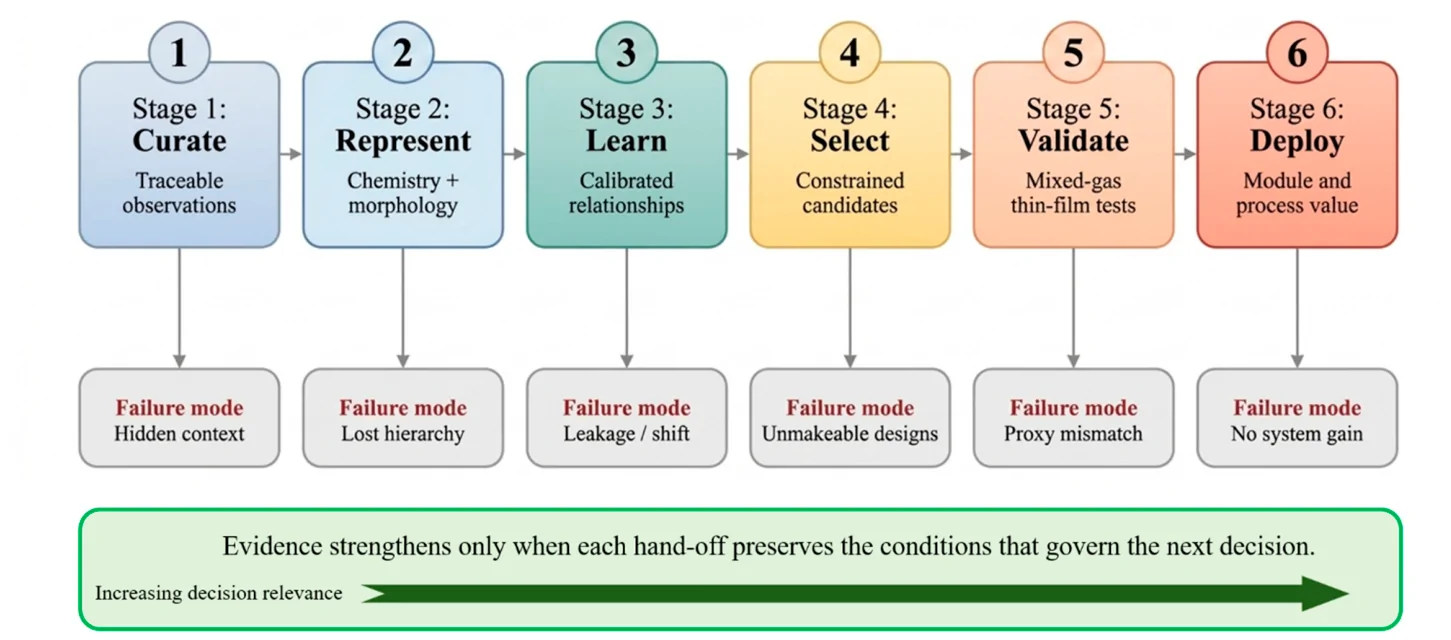
The data-to-deployment evidence pipeline for machine learning in gas-separation membranes. Each stage produces an output required by the next stage, while the lower boxes identify the characteristic failure mode.

Rather than cataloguing algorithms, we examine the hand-offs that convert a compiled membrane record into decision-relevant evidence (Figure 1). These hand-offs expose where confidence is lost: data curation can remove experimental context; representation can discard membrane hierarchy; model evaluation can leak chemical similarity; unconstrained search can generate infeasible candidates; proxy measurements can exaggerate performance; and a material-level improvement can disappear at module or process scale.

Figure 1 therefore functions as an assessment framework rather than a chronological summary of the literature. Studies are compared by the decision they enable, the origin and context of their labels, the separation between training and evaluation domains, and the strength of physical or experimental validation. A large virtual library is not treated as equivalent to a large experimental dataset, and molecular-dynamics confirmation is not treated as equivalent to membrane fabrication [31]. The evidence levels developed below formalize these distinctions and provide a consistent basis for evaluating progress across membrane classes.

To avoid any confusion between established findings and the present contribution, we state explicitly that the following elements are proposals of this review rather than conclusions drawn from a single published study: (i) the five-level evidence framework (Section 2); (ii) the contextualized observation unit and its minimum data schema (Section 3); (iii) the uncertainty-aware closed-loop discovery architecture and its acquisition formulation (Section 7); and (iv) the minimum reporting and reproducibility checklist (Section 8). Each element is introduced and justified in its corresponding section, is anchored in the cited literature where available, and is presented as a framework to be tested and refined by the community rather than as an established standard.

## 2. Evidence Levels for Decision-Relevant Membrane ML

For consistent comparison, we propose five evidence levels of increasing strength; the required level is not fixed by a universal linear scale but chosen according to the specific scientific or technological decision being addressed. This framework is our own proposal for assessing validation strength rather than a classification adopted verbatim from a single published source:

(1) Retrospective interpolation: Performance is evaluated on a random subset of records drawn from the same compiled dataset. This level is useful for software verification but remains vulnerable to material and source redundancy [29].
(2) Domain-disjoint generalization: The held-out data contain materials that are genuinely unfamiliar to the training set, such as new chemical families (e.g., new polymer repeat units or new porous frameworks such as metal–organic and covalent organic frameworks), new laboratories or new publication periods. The purpose of this level is to test transfer to unfamiliar chemistry and measurement provenance rather than interpolation among near-duplicate records. Applicability and uncertainty should be assessed on the same disjoint sets [34].
(3) Physics or simulation confirmation: Ranked candidates are checked using higher-fidelity molecular simulation, mechanistic calculations or sensitivity analysis outside the training labels [35].
(4) Prospective membrane validation: A model-selected candidate is synthesized or acquired, fabricated as a membrane and tested after selection, as demonstrated in prospective polymer studies [17,21].
(5) Deployment-relevant validation: The candidate is evaluated as a thin selective layer under mixed-gas, pressure-, humidity- and time-relevant conditions, and its contribution is examined at module or process scale [36,37].

The levels are defined around the membrane-science criteria that ultimately decide a separation, not only around statistical validation practice. Retrospective interpolation and domain-disjoint generalization establish whether a model can transfer across chemistry, laboratories and measurement provenance; physics or simulation confirmation checks consistency with transport mechanisms, such as solution–diffusion versus sorption-, diffusion- or reaction-controlled transport in the relevant pore regime; prospective membrane validation requires that the selected candidate can be synthesized, fabricated as a membrane and tested; and deployment-relevant validation introduces thin-film architecture, mixed-gas competition, aging, plasticization, defects and module- or process-level requirements. Each level therefore combines an ML validation requirement with the membrane-science criterion that a claim must satisfy before it can guide a separation decision.

These levels are not a universal linear scale that every study must climb, nor a simple score for ranking individual studies. The evidence level required depends on the specific scientific or technological decision being addressed: simulation can be the appropriate terminal evidence for a mechanistic question, and retrospective models can be valuable for data completion, provided that the corresponding claims are limited accordingly. The point is to prevent claims from outrunning their validation. Table 1 gives the resulting map of material system, representation, task and bottleneck. Because these rows synthesize established material-specific evidence rather than introduce a new taxonomy alone, the table includes a dedicated column of representative sources. It shows why a universal “best algorithm” is unlikely: the omitted variable for a polymer may be processing history, for an MOF framework flexibility, and for an MMM the interphase that connects otherwise well-described components.

Table 1 also indicates a recurring design principle: the representation should include variables that can be controlled at the decision stage. A model dominated by post hoc descriptors that are unavailable before synthesis can explain an existing dataset but cannot independently select a new experiment.

## 3. Building Trustworthy Membrane Data

### 3.1. The Observation Unit Is Not a Material Identifier

Permeability is often presented as though it were a fixed attribute of a chemical name. For dense polymer membranes, the solution–diffusion relation *P_i_* = *D_i_S_i_* shows that transport depends on penetrant sorption and diffusion [3]. Both terms are sensitive to chain packing, excess free volume, thermal history and penetrant concentration [59]. The ideal selectivity for gases *i* and *j*, *α_i_*_/_*_j_* = *P_i_*/*P_j_*, follows from the ratio of pure-gas permeabilities under matched conditions. In mixtures it is altered by competitive sorption, plasticization and coupling [13].

For thin-film composite membranes, permeance rather than permeability is frequently the experimentally relevant quantity. The apparent response includes selective-layer thickness, substrate resistance and defects [32]. Phase-separation history and manufacturing route can therefore alter transport independently of the nominal repeat unit [39,40]. Simulation-guided membrane design reaches the same conclusion because a structural model remains conditional on the membrane state [60]. For porous crystals, framework flexibility changes diffusion barriers [61], whereas missing linkers, grain boundaries and membrane microstructure introduce additional state variables [44,45]. MOF membrane reviews document how these variables enter gas-separation experiments [62]. Throughout this review, permeability denotes the thickness-normalized transport coefficient of a membrane material, whereas permeance denotes the area-normalized flux of a specific membrane architecture; pure-gas and mixed-gas measurements are treated as distinct evidence classes and are never pooled without explicit declaration.

A first step toward this record is to distinguish material-level properties from membrane-level characteristics. Material-level properties belong to the chemistry itself—repeat-unit structure, molecular mass, ideal-crystal geometry or the intrinsic transport coefficients of a defect-free bulk phase—and are, in principle, reproducible from the material alone. Membrane-level characteristics arise only when that material is processed into an actual membrane: the realized thickness, morphology and defect population, phase-separation and thermal history, support and interface state, aging and conditioning, and the resulting permeance and mixed-gas response. A model built only on material-level descriptors can rank chemistries, but it cannot predict what a fabricated membrane will deliver unless membrane-level characteristics are represented; conversely, membrane-level measurements without material identity cannot be transferred to new chemistries. Both levels must therefore be kept explicit in the observation record (Table 2).

**Figure 2:**
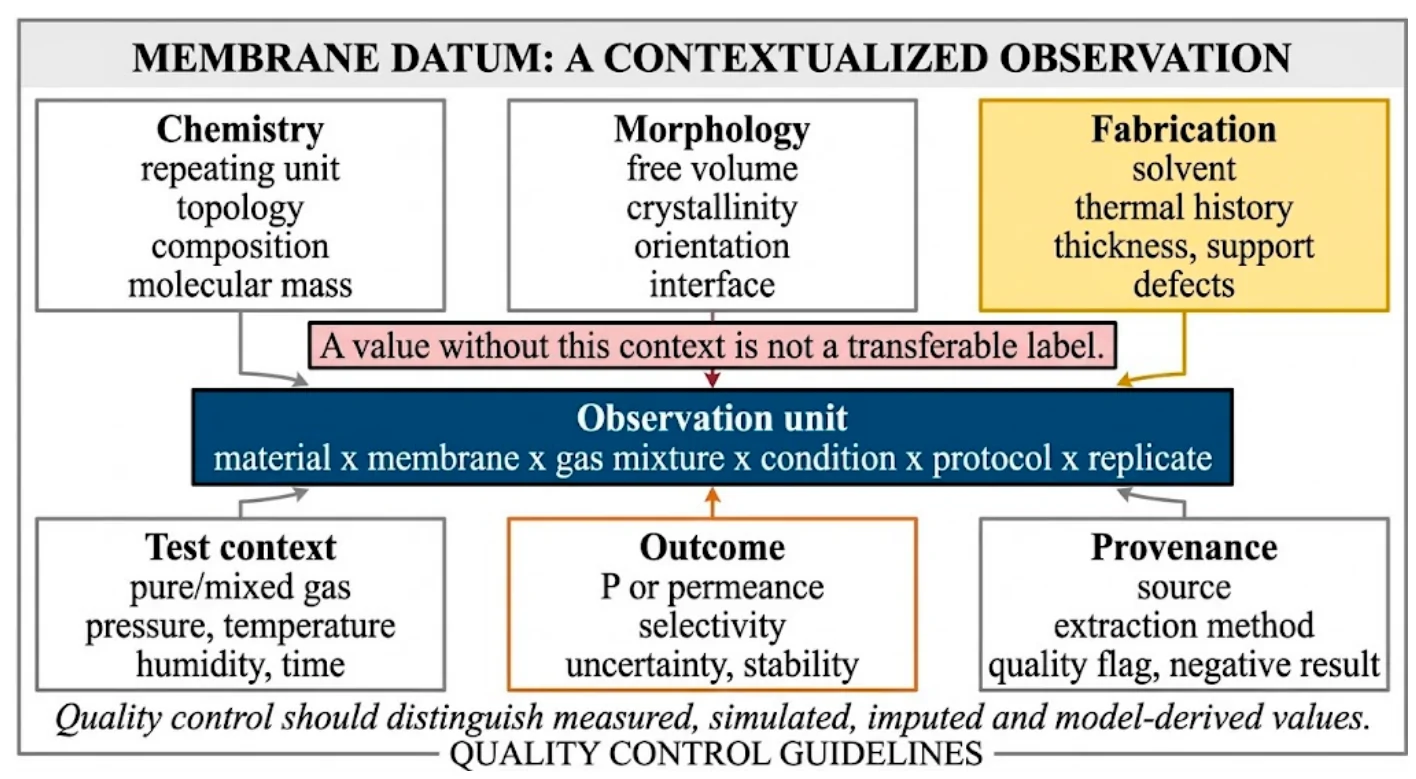
Contextual data schema for a gas-separation membrane observation. The central unit prevents chemical identity, membrane state and test protocol from being collapsed into a single decontextualized label.

Consequently, we propose that the smallest reusable record is not “material–gas–permeability” but a contextualized observation linking material identity, membrane state, mixture, operating condition, protocol, replicate and provenance; this unit is proposed in the present review and is directly motivated by the state-dependent transport arguments above. Figure 2 defines this observation unit. Its six information blocks should be stored together or explicitly marked as missing. A record with a precise permeability but no pressure, temperature, membrane age or film thickness may be less useful for transfer than a lower-precision record with complete context.

The most important implication of Figure 2 is that “metadata” are causal context, not administrative decoration. If thick and thin films age at different rates, thickness is a model input and a stratification variable. If permeability is back-calculated from permeance using a nominal thickness, the derivation belongs in provenance. If a value is imputed, simulated or produced by an effective-medium equation, it must not be silently pooled with direct measurement. Otherwise, a model can learn the method used to create the label and appear to learn membrane physics.

### 3.2. A Minimum, Quality-Controlled Data Schema

Table 2 translates the concept into a practical minimum schema. The fields are grouped according to when they become available. This separation supports two legitimate model types: design-time models, which use only information available before an experiment, and diagnostic models, which may use characterization collected after fabrication. Mixing the two without declaration creates target leakage and unrealistic claims of screening capability, particularly when repeated materials or derived descriptors cross the split boundary [29].

The schema makes negative and censored information useful. A brittle film, failed coating, phase-separated MMM or permeability below an instrument threshold is a scientific outcome, not an empty cell. Excluding such outcomes biases a model toward successful fabrication and can make generative search systematically overconfident [63]. Polymer-dataset studies illustrate the value of structured gas panels [38], while simulation and experiment data fusion demonstrates why label origin and fidelity must remain explicit [35]. FAIR principles provide a governance foundation, but membrane data also require domain-specific ontologies for gas order, membrane architecture, test mode and provenance [33].

### 3.3. Missingness, Conflicts and the Role of Imputation

Gas-permeability matrices are sparse because most studies report only a subset of gases and because very low permeabilities may be censored by instrumental limits. Missingness is therefore not automatically random. It can encode study objectives, measurement feasibility or selective reporting. Yuan et al. [16] demonstrated that ML can impute missing polymer permeability values and recover more complete performance maps. Simulation and experiment data fusion provides a complementary route when source fidelity is retained explicitly [35].

Imputation is valuable when its uncertainty is propagated and the estimates are used for hypothesis generation. It becomes hazardous when imputed labels are treated as independent measurements, used in both training and evaluation, or repeatedly re-imputed across model generations. A robust database should preserve the observed/imputed mask, the imputation model and version, the training data lineage and the prediction interval [16].

Conflicting values should not automatically be averaged. Large differences may encode pressure-dependent plasticization or thickness-dependent aging [30]. Residual solvent, phase-separation history and sample preparation can create additional variation [39,59]. The first response should be record linkage, in order to determine whether values represent the same chemical batch, membrane state and test condition. Only replicated, condition-matched observations justify statistical aggregation. When context is incomplete, hierarchical models can retain source-level or laboratory-level effects rather than assuming that all records are exchangeable. This treatment also preserves provenance and prevents a laboratory signature from being mistaken for a material effect [25,33].

### 3.4. Bias in the Accessible Literature

Published datasets over-represent high-performing membranes, familiar gas pairs, pure-gas tests and successful syntheses. They under-represent negative results, industrial feed impurities, mechanically failed films and candidates that cannot be processed below micrometer thickness [63]. Porous-framework data add a second bias because virtual libraries contain idealized structures whose synthesis, activation and stability are uncertain. Database diversification exposes this coverage problem [64], while stability-aware screening shows that candidate ranking changes when a practical constraint is included [65]. Polymer virtual libraries likewise omit polymerization feasibility and accessible molecular mass [31]. The practical consequence is asymmetric uncertainty: a model may be most confident in regions that resemble historically publishable successes rather than in regions that are genuinely low risk.

Three remedies are immediately available. First, evaluation sets should be stratified by publication time, source and chemical family because redundancy control can reveal inflated interpolation scores [29]. Second, candidate utility should include feasibility and calibrated uncertainty, not only predicted transport [34]. Third, community repositories should accept failed fabrication and stability outcomes with structured reasons [63,66]. These changes would improve discovery even without a new algorithm.

## 4. Physics-Aligned Representation and Learning

### 4.1. Represent the Hierarchy That Controls Transport

The representation is the model’s scientific hypothesis about which differences matter. Molecular fingerprints can capture functional groups and local topology in polymers, but they do not by themselves encode molecular-mass distribution, sequence uncertainty, packing frustration, aging or process history. SMILES and extended-connectivity fingerprints established widely used molecular encodings [67,68]. Polymer informatics subsequently extended representation to repeat units and macromolecular structure [69,70]. Reviews of data-driven polymer design summarize the resulting descriptor hierarchy [71,72]. BigSMILES provides a syntax for stochastic polymers [73], while ensemble graph representations extend the prediction unit beyond a single small molecule [74].

Transfer across sparse polymer-property tasks can be improved through multitask learning [75] and transformer representations [76]. General molecular GNN architectures show how three-dimensional or fingerprint information can be integrated [77,78]. Polymer-specific discussions nevertheless emphasize limited labels and out-of-domain chemistry [70,79,80]. Polymer language models provide further scale, but their value still depends on the data and task used for pretraining [81,82]. For gas separation, a chemically expressive representation is insufficient unless membrane state and test context are integrated into the model.

For MOFs and COFs, crystal graphs and pore descriptors are natural because adsorption and diffusion depend on chemistry and three-dimensional geometry. Data-driven diffusion models combine geometric and chemical variables [41,83]. Molecular-modeling surrogates accelerate larger screening spaces [42,84], while generative reticular design converts a property surrogate into a candidate generator [85,86]. Validity and applicability control remain essential in the generated space [87]. A rigid crystallographic structure can still be an incomplete state description because flexibility can change aperture size, diffusion barriers and mixture selectivity [61]. Defects and intergrowths may then dominate a fabricated membrane. A representation can therefore be numerically expressive yet physically incomplete.

MMMs require a joint representation of polymer, filler and interphase. Treating a formulation as an unordered concatenation of a polymer fingerprint, MOF descriptors and loading assumes that the two phases preserve their bulk properties and that the interface is passive. Effective-medium equations are useful baselines, but interfacial rigidification, voids, particle size, surface functionalization and processing can reverse the expected trend [50,88]. Experimental MMM studies confirm that morphology and confinement must be represented rather than inferred from bulk phases alone [47,48]. Nanoparticle assembly can also move the realized permeability–selectivity balance [89]. For CMS membranes, the precursor is only the beginning. Pyrolysis temperature, soak time, atmosphere, heating rate and thickness create the final ultramicropore distribution [51,52]. In both systems, the material is a process-dependent object.

**Figure 3:**
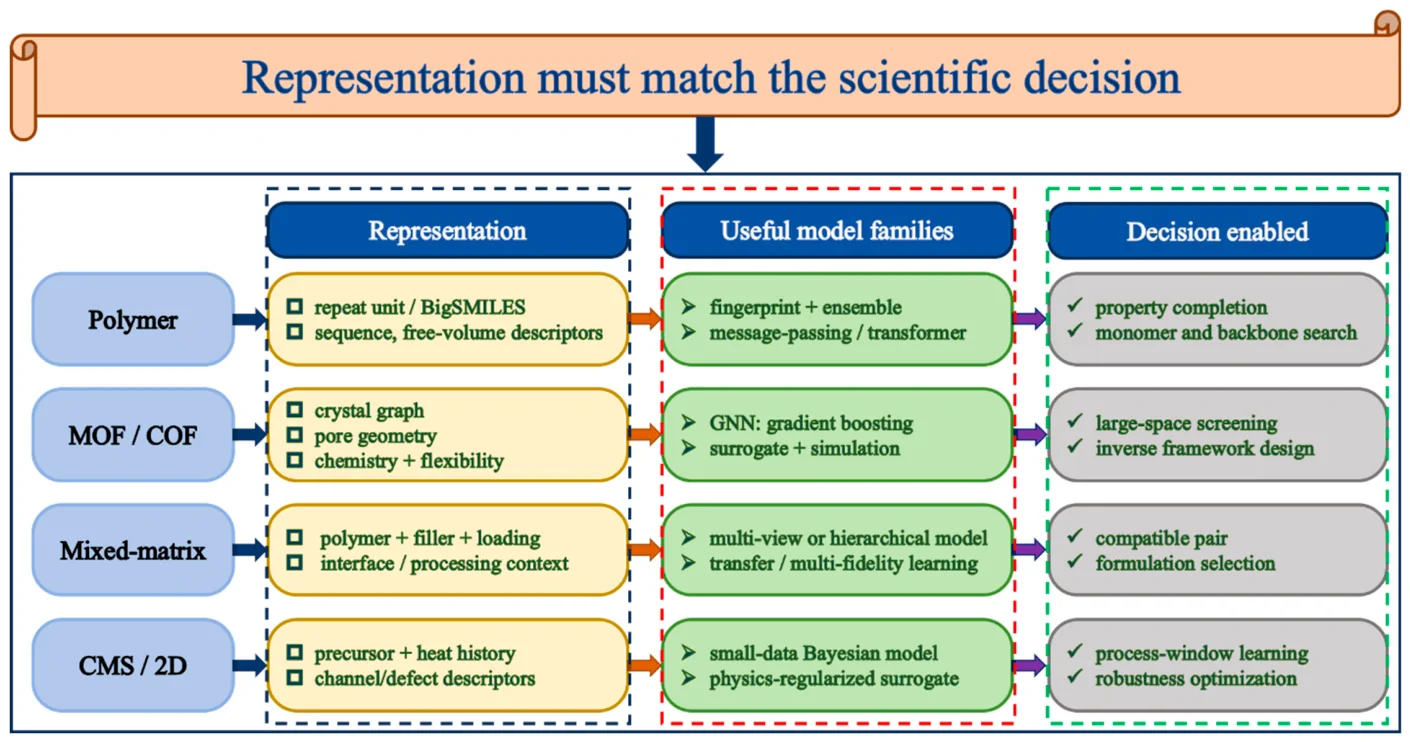
Material-aware map linking representation, model family and decision in membrane ML.

Figure 3 maps these representations to useful model families and decisions. The figure should not be read as an algorithm leaderboard. Ensemble trees remain strong for small tabular datasets, whereas GNNs are suitable when graph connectivity should be learned. Gaussian-process and Bayesian models are attractive for small, expensive experimental campaigns, while neural surrogates can accelerate large simulation spaces. Benchmark studies show that apparent model superiority depends on the split and sample size [90]. Feature-selection results also change when descriptors are redundant [91]. Interpretability reviews further caution that a plausible explanation does not compensate for an unsuitable validation design [92]. The governing criterion is whether the model’s inductive bias, uncertainty behavior and data demand match the decision.

### 4.2. Multitask, Multi-Fidelity and Physics-Informed Learning

Gas permeability is intrinsically multitask because the same membrane is tested for several gases and selectivity is derived from paired properties. Learning gases independently discards correlations and can produce inconsistent predictions. Multitask learning shares statistical strength between sparse labels in general polymer-property models [75] and gas-specific studies [18]. Simulation and experiment fusion also benefits from a multitask formulation when fidelity labels remain explicit [35]. Predicted ideal selectivity should agree with the ratio of predicted permeabilities under matched conditions, or the model should predict mixture selectivity as a separate context-dependent target.

Multi-fidelity learning is equally important. Low-cost sources include group-contribution estimates, rigid-framework screening, effective-medium equations and short molecular simulations. Higher-fidelity sources include flexible-framework calculations, long-timescale molecular dynamics, mixed-gas permeation and prospective experiments. Simulation–experiment fusion for polymer gas transport illustrates how fidelity labels can improve data efficiency without pretending that the sources are interchangeable [35]. Pooling them as if they had equal epistemic status transfers systematic error. Instead, the model should encode fidelity and learn discrepancies, or use low-fidelity predictions as priors that are corrected by measurements. This is especially relevant to MMM studies that generate tens of thousands of labels using a mixing rule: the resulting model may accurately emulate the equation without learning interfacial membrane behavior.

Physics-informed learning can enter at three levels. Features may represent free volume, pore limiting diameter, adsorption enthalpy or condensability. Constraints may enforce positivity, thermodynamic consistency, known scaling relations or monotonic trends in a restricted domain [93]. Hybrid models may learn a residual around solution–diffusion, Maxwell-type transport or a process simulator [36,94]. These choices require evidence because each constraint has a domain of validity. A constraint should be relaxed when aging, defects or non-ideal mixtures violate the baseline rather than being used only to decorate an opaque model.

### 4.3. Interpretability Is Not Causality

Feature attribution is useful for auditing whether a model relies on plausible chemistry or on spurious identifiers. Guo et al. used interpretable learning and large-scale data mining to connect MOF descriptors with predicted gas diffusivity [83]. In polymer membranes, Vigna et al. combined prediction and imputation to examine structure–property relationships for CO_2_ separation [95]. These studies illustrate the scientific value of using ML to generate testable hypotheses rather than only rankings.

Attribution nevertheless explains a model, not necessarily the membrane. Correlated descriptors can exchange importance, publication year can proxy for improved test methods, and a morphological feature measured after fabrication cannot become a causal design rule merely because it has a large SHAP value. Materials-interpretability studies recommend evaluating explanation stability and chemical validity [91,92]. Zhong et al. further emphasized that explainable ML should be used to audit and constrain materials models rather than to assert causation from a single attribution map [96].

The strongest interpretation workflow therefore combines four tests: stability across resamples, perturbation of physically controllable variables, chemically valid counterfactual candidates and prospective intervention. A feature should be promoted from correlation to design principle only when the predicted intervention survives an experiment that holds major confounders constant. This standard is especially important for membrane data because chemistry, morphology and measurement history are often correlated.

## 5. What Machine Learning Has Actually Delivered

### 5.1. Polymeric Membranes: From Imputation to Prospective Synthesis

Polymers provide the clearest progression across the evidence levels. Historical models predicted permeability from hand-crafted descriptors on relatively small datasets [14,15]. Zhu et al. later extended data-driven polymer screening [97]. These studies established learnability, but their applicability domains were narrow and membrane context was usually incomplete. Deep ensembles and multimodal models have since expanded the prediction strategies [98,99]. Practical tabular and interpretation studies remain largely retrospective [95,100]. Yuan et al. [16] then addressed the data problem of missing gas panels rather than only optimizing a benchmark score. The study illustrates a valuable role for ML as infrastructure, provided imputation is identified and uncertainty is retained.

Barnett et al. [17] moved closer to discovery. A model trained on roughly 700 measured polymers was used to predict more than 11,000 homopolymers, and two selected polymers were synthesized and tested; both exceeded the then-relevant CO_2_/CH_4_ upper bound. This prospective loop is more consequential than the screening-library size because it tests the complete chain from representation through material acquisition to permeation. It also exposes what remains: performance under mixed gases, aging, thin-film fabrication and process conditions.

Yang et al. [18] combined multitask graph learning, interpretable analysis and screening of more than nine million hypothetical polymers, followed by molecular-dynamics checks. Giro et al. [20] integrated generative monomer design, prediction and automated molecular simulation for carbon-capture polymers. Wang and Jiang [93] used a physics-informed performance metric and active learning to screen 155,610 PIM candidates while calculating only a small fraction. Basdogan et al. [101] coupled ML with a genetic algorithm for CO_2_ separations. Targeted backbone libraries provide a more constrained search space [38], while inverse-design and ensemble strategies expand it [102,103]. More recently, Yue et al. [104] constructed LadderGen, an approximately 0.8-million-member ladder-polymer library, and combined ML screening with molecular-dynamics validation. These studies show how ML can allocate computation and search combinatorial spaces, but their candidates remain hypotheses until polymerization, molecular-mass control, film formation and realistic permeation are demonstrated.

Xu et al. [21] supplied an important second example of prospective polymer validation. Explainable graph learning produced candidate polymers that were synthesized and tested, with reported upper-bound-exceeding performance for several gas pairs. Machine-learning-guided synthesis of PEO-based membranes and experimental optimization of polymer nanocomposites further demonstrate that the model–experiment hand-off is feasible beyond a single chemistry [105,106]. As with Barnett et al. [17], the crucial evidence is not simply novelty in latent space but successful hand-off to experiment. Such studies should become the reference pattern: publish the pre-specified candidate-selection rule, include failed candidates, and compare prospective error with retrospective cross-validation.

### 5.2. Porous Frameworks: Scale, Speed and the Ideal-Structure Gap

MOFs and COFs provide enormous structurally explicit libraries, making them natural targets for surrogate models. Bai et al. [107] combined high-throughput calculations and ML to screen MOF membranes for hydrogen separation. Situ et al. [108] screened 3806 MOFs for flue-gas CO_2_ capture and examined water in a ternary feed for leading candidates, a useful step beyond dry binary proxies. Daglar and Keskin [88] combined molecular simulation and ML to evaluate both MOF membranes and MOF/polymer MMMs. Reviews connecting precise MOF design to supported-membrane fabrication locate the boundary between framework ranking and membrane evidence [109]. Diffusivity models show the effect of target definition [83,84]. Acetylene-separation studies add gas-specific constraints [110,111], while stability-aware CO_2_ screening changes rankings through feasibility [65].

Inverse design expands screening into generation. Zhou and Wu [19] used a genetic algorithm to design MOFs for C_2_H_4_/C_2_H_6_ separation, while earlier work combined physics and data modeling for D_2_/H_2_ membrane separation [112]. Generative reticular models and Bayesian optimization provide complementary search strategies [85,86]. These studies show that an ML surrogate can navigate structural variables far faster than direct simulation. Yet the inverse problem is under-constrained when synthesizability, activation stability, framework flexibility, defect formation and polycrystalline membrane growth are excluded. A generated framework is therefore best described as a computational design hypothesis, not a discovered membrane.

COF studies have followed a similar pattern. Aydin et al. [113] used high-throughput screening for helium separation and hydrogen purification, including COF/polymer MMMs. Xin et al. [114] combined interpretable ML and molecular simulation for acid-gas separation. Newer campaigns have expanded the searchable COF space [115,116]. The structural regularity of COFs enables compact descriptors, but stacking disorder, pore blockage, hydrolytic stability and continuous-layer formation remain weakly represented. Experimental pore engineering identifies the importance of realized aperture chemistry [117,118]. Ultrathin-film assembly introduces thickness and support effects [119,120]. Ionic and functional COF membranes further show that charge, swelling and fabrication conditions modify ideal-crystal expectations [121,122]. Additional membrane demonstrations emphasize continuity and defect control [123,124]. For both MOFs and COFs, the next gain may come from learning the probability that a framework can be fabricated as a stable, low-defect membrane.

### 5.3. Mixed-Matrix Membranes: Learning the Interface, Not the Mixing Rule

MMMs are attractive because a processable polymer can host a high-performance porous filler, but they are among the hardest systems to learn. Guan et al. [125] used ML to predict MOF-based MMMs for CO_2_ capture, while Zhang et al. [126] considered ionic-liquid–MOF composites. Yao et al. [127] developed a separate model for CO_2_-separation performance. These studies established the value of formulation-level descriptors without resolving all interfacial variables.

Larger computational campaigns have expanded the candidate space. Wan et al. [128] analyzed 54,117 polymer–MOF combinations using interpretable ensembles. Yu et al. [94] screened 131,722 combinations and incorporated framework flexibility through a Maxwell-model layer. Experimental evidence explains why filler chemistry alone remains insufficient. Particle morphology and porous-network architecture alter the realized pathway [47,48], while polymer rigidification, confinement and non-selective voids depend on interfacial chemistry and processing [50].

Scale must be interpreted carefully. If MMM labels are generated by a Maxwell-type equation from neat-polymer and neat-filler properties, a high-accuracy ML surrogate demonstrates fast reproduction of that model [94]. It does not validate filler dispersion, polymer rigidification, non-selective voids or changes in polymer fractional free volume. The model can still eliminate clearly unfavorable pairs, but the uncertainty associated with the effective-medium assumption should be explicit.

High-value MMM datasets must therefore combine transport with particle size and loading, casting protocol, microscopy or scattering evidence, interfacial chemistry and failed-film outcomes [47,50]. The relevant prediction target is the process-created interphase and its defect state, rather than the identities of the two bulk phases alone.

### 5.4. CMS and Two-Dimensional Membranes: Promising Small-Data Regimes

CMS membranes convert a polymer precursor into a kinetically selective carbon network. Pan et al. [52] used ML to analyze variables controlling CMS gas-separation performance. A recent CMS review likewise shows that precursor chemistry and pyrolysis history must be separated to identify a transferable process window [51]. Aging and module geometry add further context [53]. This system is well suited to Bayesian optimization and active learning because each pyrolysis and test cycle is costly but controllable. It is poorly suited to naive pooling because identical nominal temperatures can produce different structures when heating rate, soak time, atmosphere, precursor history and film thickness differ. The most useful future model is likely a calibrated surrogate embedded in a standardized fabrication campaign.

For atomically thin membranes, computation can resolve pore chemistry and transport mechanisms, but realized defect distributions, supports, transfer damage and scalability control device behavior. Graphene and graphene oxide studies demonstrate the sensitivity to pore creation and interlayer spacing [129,130]. MXene membranes add surface termination, channel swelling and stacking as design variables [131,132]. Molecular-sieving studies connect those variables to gas transport [133,134]. Support choice and scale-up method can then outweigh an ideal intrinsic barrier [57,58]. A machine-learning-assisted CVD study for hydrogen-isotope sieving illustrates how synthesis control can enter the learning loop [135]. Gas-specific ML evidence nevertheless remains sparse compared with polymer and MOF work. This gap creates an opportunity for small-data, physics-constrained learning tied directly to synthesis and microscopy. Claims for this class should clearly separate ideal-pore simulation from measured membrane permeance.

### 5.5. From Material Property to Process Value

A membrane is valuable only within a separation flowsheet. Cheng et al. [36] integrated molecular simulation, an ML surrogate and process modeling for MOF-membrane CO_2_/CH_4_ separation. This study illustrates a multiscale route from material properties to a hollow-fiber design. AI-assisted equipment optimization addresses operating variables at a second scale [136]. Hybrid-process superstructures compare membranes with complementary unit operations [137], whereas application-specific process models expose separation-dependent constraints [138,139]. Such integration reveals that maximizing selectivity alone may increase area, compression duty or pressure-ratio sensitivity, while maximizing permeability (or, for thin-film architectures, permeance) may sacrifice purity or hydrocarbon recovery. The appropriate objective is application specific and typically includes energy, membrane area, productivity, purity, recovery, replacement interval and carbon intensity.

Table 3 compares representative gas-separation-specific ML studies using the evidence framework. The table is deliberately selective rather than exhaustive and emphasizes label origin and the strongest validation step. Every row summarizes a primary study, so the numbered reference is retained in the rightmost column. Two patterns stand out. The largest search spaces are mainly supported by simulation or mixing-rule labels. Prospective experimental validation exists but remains concentrated in polymer studies and rarely reaches mixed-gas thin-film or module conditions.

Table 3 supports a more precise conclusion than “ML accelerates membrane discovery.” ML has already accelerated data completion, simulation and candidate prioritization. Evidence that it routinely accelerates fabricated, durable and process-relevant membranes is much thinner. That gap should determine how future studies are designed and how claims are worded.

Viewed through the four membrane-science dimensions, the surveyed advances connect as follows. For reproducible fabrication, ML has contributed mostly to candidate selection and process-window identification, whereas reproducible fabrication itself—controlled casting, crosslinking, pyrolysis and defect minimization—remains largely protocol-dependent manual work that ML has only begun to assist [51,52,63,66]. For characterization, ML has accelerated the interpretation of transport and structure-property data [83,95], but standardized, machine-readable characterization records are still scarce. For realistic gas transport measurements, progress is strongest in pure-gas and simulated-mixture regimes, while mixed-gas, humid and contaminant-containing measurements remain rare in ML datasets (Table 3). For durable membranes, aging, plasticization and stability outcomes are almost absent from ML training and evaluation, so durability claims remain prospective rather than demonstrated. This mapping shows that ML’s demonstrated value currently concentrates in screening and data infrastructure, whereas the fabrication-to-durability chain still requires the coordinated advances prioritized in Section 9.

## 6. The Validation Gap

Much of this gap originates in membrane science itself, not only in statistical practice. Fabrication is protocol-dependent: casting solvent, evaporation and annealing times, crosslinking and thermal history can change the permeability of the same chemistry by orders of magnitude, so a model validated on records that omit these variables will fail whenever the membrane state differs. Morphology couples the same chemistry to different transport outcomes—crystallinity, free-volume distribution, chain orientation or filler alignment—so descriptors that encode only chemical identity cannot transfer across morphologies. Defects, including pinholes, grain boundaries, interfacial voids and cracks, can collapse selectivity far below the value predicted for the ideal structure, which explains why computation-validated candidates often underperform in experiment. Physical aging, plasticization and swelling shift permeability and selectivity over time and under mixed feeds, so a validation performed at a single point in time cannot certify long-term behavior. Finally, the operative transport mechanism—solution–diffusion, pore-controlled transport or facilitated transport—determines which variables matter, and a model that implicitly assumes one mechanism cannot be expected to validate across another. These membrane-science sources are at least as important as data-splitting and uncertainty issues in creating the validation gap; they are also the reason why the evidence framework of Section 2 terminates in deployment-relevant testing rather than in prediction accuracy. The data-splitting, uncertainty and testing-condition aspects are discussed in Section 6.1 and Section 6.2.

### 6.1. Similarity Leakage and Domain Shift

Random train/test splitting answers a narrow question: can the model interpolate records drawn from the same collection? It is a useful software check but a weak discovery test, as broader materials benchmarks demonstrate when redundancy is removed [29]. Repeat units with minor substitutions, MOFs sharing a topology or linker family, multiple gases for the same membrane, and records extracted from one publication can appear on both sides of a split. Membrane-ML reviews have identified this mismatch between record-level accuracy and material-level generalization [25,26]. If data augmentation, imputation or descriptor normalization is performed before splitting, information can leak more directly. The apparent test set is then statistically held out but scientifically familiar.

At minimum, polymers should be split by scaffold or similarity cluster, porous frameworks by topology, linker, metal cluster or structural cluster, and MMMs by polymer–filler family. All measurements of one membrane specimen should remain in one fold. Source-disjoint splits test transfer across laboratories and extraction conventions, while temporal splits mimic use on future literature. A truly external dataset tests the whole curation and prediction pipeline. Redundancy control can change model ranking and error sharply [29]. Under domain shift, the associated uncertainty must also be assessed because a similar mean error can conceal poorly calibrated intervals [34]. For membrane datasets, these changes quantify how much apparent accuracy came from local familiarity.

**Figure 4:**
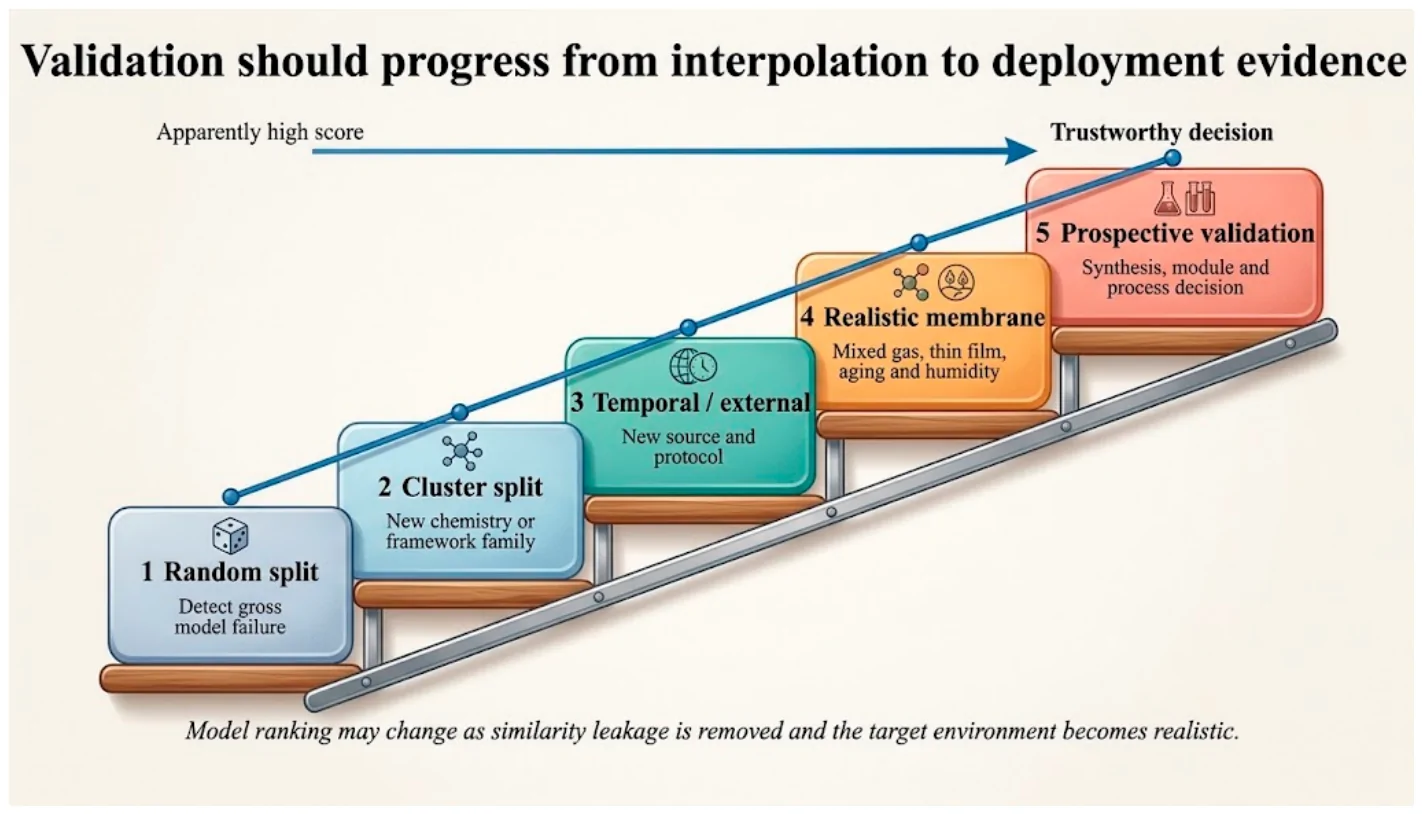
Validation ladder for membrane ML.

The validation ladder in Figure 4 formalizes this progression. Movement up the ladder typically lowers the apparent score because each step removes a source of familiarity or adds physical realism. At the same time, decision confidence increases. The correct response to a lower chemistry-disjoint score is not to retreat to a random split; it is to narrow the applicability domain, improve representation or collect informative data. As with the evidence levels in Section 2, the appropriate rung of this validation ladder depends on the specific decision being addressed.

### 6.2. Pure Gas, Thick Films and Equilibrium Proxies

Pure-gas permeability measured on a thick, defect-free film is an efficient characterization tool, but it can be a poor proxy for an industrial membrane. Competitive sorption changes mixture selectivity, while CO_2_ and hydrocarbons can plasticize glassy polymers [140]. Water may compete for sorption sites or alter framework flexibility. Strongly sorbing impurities can also block transport.

Thin selective layers often age more rapidly, contain a different packing state and amplify the effects of pinholes and support resistance [30,140]. Plasma modification changes the realized transport response [141], as do hybrid fillers [142] and atomic-layer-deposition tuning [143]. The distinction between permeability and permeance is therefore decisive. Pure-gas upper bounds cannot by themselves establish mixed-gas process performance [144].

Model evaluation should match the intended application. A natural-gas model needs high-pressure CO_2_/CH_4_ mixtures, relevant hydrocarbons, water and time-dependent plasticization. A post-combustion capture model needs low CO_2_ partial pressure, water and trace contaminants, as well as thin-layer permeance. An H_2_-purification model must define temperature, pressure and competitive species. Fabrication-oriented analyses require processing variables to be part of the model scope [63]. Nanocomposite guidelines make the same point for geometry and interface state [66], while hollow-fiber studies show that module architecture changes the usable target [145]. If these labels do not exist, the claim should be limited to pure-gas screening and the uncertainty associated with transfer should be stated.

### 6.3. Performance Is Time Dependent

Physical aging, plasticization and chemical or morphological instability convert a static prediction into a trajectory. High-free-volume polymers can lose permeability as nonequilibrium free volume relaxes, and thin films may age on a different timescale from bulk films [30]. Manufacturing reviews confirm that thickness and treatment history must accompany transport data [32]. CMS structures can evolve with oxygen exposure or temperature [51]. MOF membranes respond to water, pressure cycling, framework flexibility and defects [61,146]. A single early-time value may therefore select a candidate that becomes inferior after conditioning.

A reusable record should include conditioning protocol, measurement time and the direction and rate of performance change. Models can then predict parameters of an aging or plasticization curve, survival above a performance threshold, or robust utility integrated over service time [30]. Repeated measurements should not be randomly separated across folds because that leaks the membrane-specific trajectory. Prospective studies should predefine a minimum duration or stabilization criterion rather than reporting the most favorable point.

### 6.4. Uncertainty, Calibration and Applicability Domain

Discovery decisions require both expected performance and the probability of being wrong. Deep ensembles, Gaussian processes, conformal prediction, quantile regression and Bayesian neural networks can supply predictive intervals, but interval width alone is not enough. Calibration should be checked so that nominal coverage agrees with empirical coverage on chemistry-disjoint and external sets. Bayesian optimization provides a formal link between uncertainty and the next experiment [147]. Methodological reviews explain the exploration, exploitation and stopping trade-offs [148], while active-learning surveys clarify how selection bias accumulates during sequential sampling [149]. Polymer-informatics benchmarking confirms that uncertainty methods differ materially under domain shift [34].

Uncertainty has at least three sources: aleatoric uncertainty reflects measurement variability and unresolved conditions; epistemic uncertainty reflects sparse training coverage and model uncertainty; and structural uncertainty reflects omitted physics or a wrong proxy, such as using rigid-framework simulation for a flexible, defective membrane. More data of the same type may reduce epistemic uncertainty but will not correct structural mismatch. UQ benchmarks show that methods with comparable average accuracy can have different calibration behavior [34]. Model cards should therefore pair intervals with an applicability-domain indicator, distance to training chemistry, label origin and explicit caveats about the target condition [96].

Candidate selection should optimize an uncertainty-aware utility rather than the mean prediction. Depending on the purpose, this may be a conservative lower confidence bound, expected process value, probability of meeting a multi-attribute specification, or expected information gain [150]. Reporting only the highest predicted permeability systematically favors extrapolative and overconfident candidates, particularly outside the training domain [34].

### 6.5. Experimental Validation Must Test the Selection Rule

A prospective experiment is strongest when it tests the complete, pre-specified workflow. The candidate list, feasibility filters, acquisition function, target conditions and stopping rule should be fixed before results are known. Failed syntheses, insoluble polymers, cracked films and unfavorable measurements should remain in the reported denominator. Choosing a convenient candidate after screening and presenting only the successful experiment validates that specimen, not the selection policy.

Two additional controls are valuable. First, compare the ML-selected candidates with expert-, diversity- and simple-baseline selections under the same experimental budget. Second, use a blinded or time-stamped holdout when possible. These controls separate gains from the model from gains due to a larger candidate inventory, post hoc choice or increased experimental effort. Barnett et al. [17] and Xu et al. [21] demonstrate the feasibility of prospective polymer validation; the field now needs the same discipline for MMM processing, porous-crystal membranes and thin-film composite architectures. Large-scale multilayer fabrication and sustainable solvent selection show why the prospective test must include a credible manufacturing route, not only a transport coupon [37,151].

## 7. From Screening to a Closed-Loop Discovery System

### 7.1. The Loop Should Optimize a Membrane Decision

Active learning selects the next experiment to reduce uncertainty or improve an objective, and it is well suited to expensive materials campaigns [150]. Closed-loop demonstrations show how model selection can be linked to synthesis and characterization [152]. AI and high-performance computing broaden this architecture to larger materials workflows [153], while autonomous-laboratory studies demonstrate the need for explicit experimental state and feedback [154]. For gas-separation membranes, a generic autonomous loop is still insufficient. Polymer synthesis requires route planning and molecular-mass control, film fabrication contains tacit process knowledge, mixed-gas testing is slow, and a candidate can fail mechanically before transport is measured. The loop must therefore be constrained, staged and supervised by domain expertise.

**Figure 5:**
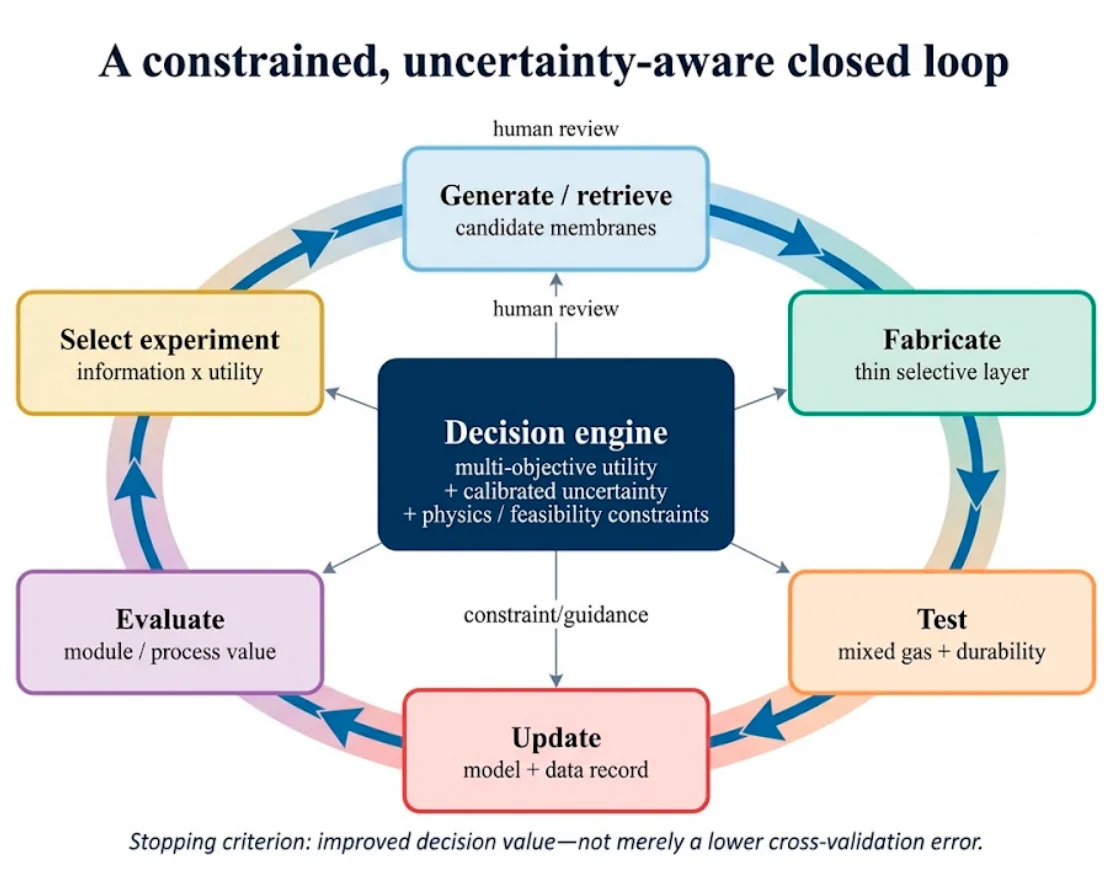
Constrained, uncertainty-aware closed loop for gas-separation membrane discovery.

Figure 5 outlines the constrained, uncertainty-aware closed-loop architecture proposed in this review, which synthesizes established active-learning and autonomous-experiment principles [150,152,153,154] rather than reproducing a single published design. Candidate generation or retrieval feeds a decision engine that combines predicted process utility, calibrated uncertainty, physical constraints and feasibility. The selected experiment is fabricated as the relevant membrane architecture, tested under informative gas conditions, and evaluated at material and process levels. All outcomes, including failures, update both the dataset and the model. Human review appears within the loop because feasibility, safety, measurement diagnostics and unexpected morphology are not fully encoded.

The key distinction in Figure 5 is the stopping criterion. A loop should stop when the expected value of another experiment is small, a pre-defined membrane/process requirement is achieved, or the remaining uncertainty cannot be reduced with the available measurement. It should not continue merely to improve a retrospective score. This makes the experimental budget and decision boundary part of the method.

### 7.2. Feasibility and Manufacturability as Learned Outcomes

Feasibility should be represented probabilistically rather than as a final manual filter. For polymers, outcomes include monomer availability, reaction success, attainable molecular mass, solubility, film formation, glass-transition or decomposition window, and crosslinking [31,38]. For MOFs and COFs, they include synthesis, activation, water and chemical stability, crystal size, grain-boundary control and supported-layer growth [65,146]. For MMMs, they include colloidal stability, viscosity, dispersion, adhesion and defect-free coating [50]. A candidate with slightly lower predicted transport but a high probability of reproducible fabrication may have higher experimental and process utility.

This formulation also makes negative data valuable. A failed polymerization updates synthesis feasibility, a cracked film updates processing risk, and non-selective voids update compatibility. Fabrication-focused ML studies identify these outcomes as missing targets rather than nuisance records [63,66]. Multi-output models can learn transport and failure modes jointly, while censored-likelihood models can retain below-detection and unsuccessful outcomes. Over time, the loop learns both which chemistry performs and which route produces a testable membrane.

### 7.3. Multi-Objective and Process-Aware Acquisition

The acquisition function should represent the application. As shown in Equation (1), a review-level utility can combine expected process value with penalties for uncertainty, experimental cost and fabrication failure. This expression synthesizes constrained active-learning and Bayesian-optimization principles rather than reproducing a single published equation [147,150].

(1) *U*(*x*) = 𝔼[*V*_process_(*x*)] − *λ_u_* *R*_uncertainty_(*x*) − *λ_c_* *C*_experiment_(*x*) − *λ_f_* *R*_failure_(*x*)

where *V*_process_ may combine energy, area, purity, recovery, productivity, lifetime and carbon intensity. The uncertainty, cost and failure terms make the choice budget-aware. For exploratory campaigns, an information-gain term can be added according to established Bayesian-optimization strategies [148]. For deployment, a conservative probability of meeting the full specification may be preferable, and its calibration should be verified under domain shift [34].

Linking Equation (1) to a process surrogate prevents misleading material optimization. The relative value of permeability and selectivity depends on feed pressure, stage configuration, compression, recycle and product constraints [136]. A process model also exposes diminishing returns. Beyond a certain permeability, or beyond the corresponding permeance for thin selective layers, support or mass-transfer resistance may dominate, while additional selectivity may produce negligible energy savings. Cheng et al. [36] offer an early illustration of this multiscale connection. Future work should propagate calibrated uncertainty through the process model rather than evaluate point estimates alone [34].

### 7.4. A Staged Experiment Sequence

A staged sequence follows naturally from active learning because each measurement should justify the cost of moving to a more realistic fidelity [35,150]. The sequence begins with inexpensive feasibility evidence and promotes candidates only when the preceding uncertainty has been reduced sufficiently:

(1) Feasibility screen: Structure validity, synthesis route, stability and processability.
(2) Low-cost characterization: Density, glass transition, porosity, film integrity and single-gas permeance.
(3) Mechanism-discriminating tests: Pressure series, sorption/diffusion separation, competitive mixtures and humidity.
(4) Application validation: Thin-film composite or hollow-fiber fabrication, aging/plasticization and contaminants.
(5) Module/process evaluation: Reproducibility, scale-up, area/productivity, energy and techno-economic sensitivity.

Each stage should have explicit promotion criteria. This prevents expensive mixed-gas testing of candidates that fail basic film formation while ensuring that apparent single-gas winners do not bypass realistic conditions. The final stages should use a credible thin-film or hollow-fiber manufacturing route [32,37] and connect the measured response to module or process requirements [36].

## 8. Minimum Reporting and Reproducibility Standard

ML papers in membrane science often report model architecture and a coefficient of determination but omit the information needed to reproduce the scientific split, trace a value or judge deployment relevance [25,26]. Table 4 provides the minimum evidence and reporting checklist proposed in this review, aligned with the pipeline. The checklist is intentionally model agnostic and applies to neural networks, ensembles, Gaussian processes and symbolic models.

Table 4 should be used at project design, not only at manuscript submission. The split and validation level must be decided before model tuning. A source-disjoint test created after many random-split experiments is still susceptible to methodological overfitting. Similarly, a model is not reproducible if the code is public but the mapping from each label to its source, condition and transformation is unavailable. Fabrication and test protocols should be treated as reproducible model inputs rather than supplementary narrative [63,66].

Metric choice should follow the decision. Mean absolute error on log permeability is interpretable for multiplicative errors; root-mean-square error emphasizes large misses; Spearman or top-k recall may be relevant to screening; and classification metrics apply only after a scientifically justified threshold is defined. Multi-objective screening should report recovery of the Pareto set or probability of meeting a specification, not separate gas-wise R^2^ values alone. Every reported interval should include calibration or empirical coverage.

## 9. Research Priorities and Outlook

The field does not need an indiscriminate expansion of model size. It needs coordinated advances at the points where evidence is currently lost, a conclusion also emerging from recent membrane-ML reviews [25,26]. Table 5 prioritizes actions by time horizon and specifies a measurable outcome. Near-term items focus on data and evaluation infrastructure. Medium-term items connect prospective experiments to thin-film and mixture conditions. Long-term items establish distributed, process-aware discovery.

### 9.1. Community Benchmarks Should Be Hard by Construction

A useful benchmark must prevent the shortcuts that dominate random splits. Each record should have a material group, source group, time stamp, label origin and test-context completeness score. Redundancy-controlled studies show why material-level grouping must be enforced [29]. Test sets should include new scaffolds or framework clusters, realistic mixtures and a small prospective component. Performance should be reported for interpolation and OOD tasks separately. A benchmark should also include a “do not predict” option so that calibrated abstention is rewarded when a candidate lies outside the domain [34].

For polymers, benchmark tasks could include completion of a gas panel, chemistry-disjoint pure-gas prediction, transfer from thick to thin films and prediction of aging trajectories [16]. For MOFs and COFs, tasks should separate ideal rigid simulations, flexible or defective simulations and measured membrane data [61]. For MMMs, at least one split should hold out both a polymer family and a filler family, forcing the model to learn transferable interface and processing relationships rather than memorize combinations [50].

### 9.2. Prospective Challenges Should Count Every Attempt

Prospective community challenges can transform the evidence base. A shared candidate pool and target condition would allow teams to submit a ranked list with uncertainty and feasibility. Independent laboratories could then synthesize or fabricate a pre-declared number of candidates. Existing prospective polymer studies demonstrate that model-selected candidates can be synthesized and tested [17,21]. A community challenge should extend this standard by reporting success rate, cost, calibration, thin-film permeance, mixture selectivity and stability in addition to the best observed sample.

The experimental denominator is essential. If ten candidates are proposed and one is reportable, the result differs from a one-shot success even when the best membrane is identical. Failed attempts should update the benchmark and become training data for the next round, consistent with active-learning practice [150].

### 9.3. Data-Sharing Models Must Accommodate Industrial Reality

Industrial membrane data are valuable precisely because they contain realistic formulations, defects, modules and contaminants, but raw disclosure may be impossible. Federated learning, secure aggregation and shared evaluation containers could allow models to learn across organizations without centralizing proprietary records. The community can begin with lower-risk shared elements such as common ontologies, reference membranes, test protocols, unit and metadata validators, and challenge test sets maintained by a neutral body. FAIR principles provide the shared-data foundation even when records cannot all be centralized [33].

Data quality should remain visible in every model. A prediction trained mostly on simulated ideal structures should not acquire the same evidential status as one supported by replicated mixed-gas thin-film measurements. Provenance-weighted learning and model cards can make that distinction operational [96,155].

### 9.4. The Scientific Payoff: Testable Mechanisms, Not Only Rankings

Decision-grade ML can contribute to membrane science as well as discovery. Interpretable MOF models demonstrate how learned diffusivity relationships can generate mechanistic hypotheses [83]. Polymer studies have similarly used ML as a microscope for structure–property relationships [95]. A model that separates chemistry, morphology, process and test context can then propose interventions, such as varying annealing at fixed chemistry or interface treatment at fixed composition. Uncertainty can identify experiments that distinguish sorption-controlled and diffusion-controlled hypotheses. If these predictions are tested prospectively, explainability becomes a route to mechanism rather than a retrospective visualization [96].

The most durable contribution of ML may therefore be a better experimental design language: which membrane state is being predicted, under which mixture and history, with what uncertainty, and which next experiment can falsify the model. This language is more valuable than another algorithm taxonomy [92].

## 10. Conclusions

ML has moved gas-separation membrane research beyond simple permeability regression. It can complete sparse property matrices, learn across gases, accelerate molecular simulation, screen combinatorial polymer and framework spaces, support inverse design, and, in notable polymer studies, select candidates that survive prospective synthesis and permeation testing. These are substantive achievements. They do not yet establish routine discovery of manufacturable, durable and process-superior membranes.

The decisive bottleneck is the evidence chain. A membrane datum must bind chemistry to morphology, fabrication, gas composition, operating condition, time and provenance. Representation must preserve the hierarchy relevant to the material class. Validation must remove chemical and source similarity before introducing mixed-gas, thin-film and temporal realism. Uncertainty must be calibrated under domain shift. Candidate selection must include feasibility and process utility. Finally, prospective experiments must disclose the full selection rule and every attempted candidate.

Organizing the field around this chain changes the research priority. The near-term opportunity is not simply to enlarge models or virtual libraries; it is to create context-rich datasets, hard benchmarks and prospective challenges. The medium-term goal is an uncertainty-aware, multi-fidelity loop that learns from failed fabrication as well as successful transport. The long-term goal is a distributed discovery infrastructure linking chemistry, membrane manufacturing, realistic permeation and process design.

We therefore propose “decision-grade ML” as an emerging evaluative framework for machine learning in membrane science: a calibrated model that knows its domain, preserves physical context, proposes feasible experiments and demonstrates value at the condition where the membrane will be used. Under this framework, ML is neither a replacement for membrane physics nor a decorative prediction layer. It is a disciplined instrument for deciding which hypothesis to test next and for learning honestly from the result.

## Figures and Tables

**Table 1:** Material-specific mapping from representation to decision and dominant validation bottleneck.

Material System	Representation and High-Value ML Decision	Dominant Bottleneck and Decisive Evidence	Reference
Solution-processable polymers	Repeat unit/sequence plus molecular mass, packing, fabrication and test context; complete sparse gas panels and rank feasible backbones	Chemistry is confounded with morphology, aging and protocol; require chemistry-disjoint prediction followed by prospective thin-film mixed-gas testing	[6,32,35,38,39,40]
MOFs and COFs	Crystal/reticular graph, pore geometry, chemistry, flexibility and stability; accelerate diffusion/selectivity screening and inverse design	Idealized rigid, defect-free structures dominate; require flexible/defective models plus fabricated-membrane and realistic-mixture validation	[7,41,42,43,44,45]
MMMs	Joint polymer–filler–interface representation with loading, particle size and processing; select compatible pairs and formulations	Sparse, non-standard interface data; require imaging/defect descriptors and prospective fabrication across laboratories	[9,46,47,48,49,50]
CMS membranes	Precursor, thickness and complete pyrolysis history plus pore descriptors; learn process windows and gas-pair response	Small datasets and irreversible protocol dependence; require Bayesian/active learning in controlled campaigns with durability tests	[51,52,53]
Two-dimensional/atomically thin membranes	Lattice/pore chemistry, defect distribution, support and transfer state; screen pores, defects and synthesis windows	Ideal-pore calculations do not represent scalable films; require coupled synthesis–characterization–permeation loops on supported membranes	[10,54,55,56,57,58]

**Table 2:** Minimum data schema for reusable ML studies of gas-separation membranes.

Block	Required Fields	Strongly Recommended Fields	Quality Control/Encoding
Identity	Persistent material ID; chemical structure or precursor; material class; composition	Molecular mass/dispersity; batch; framework database identifier; filler surface treatment	Canonical structure plus raw representation; versioned identity mapping
Membrane fabrication	Architecture with explicit geometry (dense, asymmetric, hollow-fiber or thin-film composite), fabrication route and selective-layer thickness; for thin-film systems, coating method and substrate type; support; key solvent; thermal treatment	Casting concentration; evaporation and annealing times; crosslinking (agent, degree and method, e.g., thermal, chemical or radiation-induced); filler loading/size; pyrolysis atmosphere and ramp	Units and method; distinguish measured from nominal thickness; protocol ID
Morphology/state	At least the measurements used as model inputs, with the measurement method and quantitative metric specified	Density, free volume, crystallinity, porosity, orientation, interfacial and defect metrics	Instrument/method; replicate; uncertainty; prohibit unavailable-at-design-time features in screening claims
Feed and conditions	Gas species and composition; pure or mixed gas; upstream/downstream pressure; temperature; measurement mode	Humidity, contaminants, stage cut, exposure time, preconditioning	Standard units; partial pressures; equilibrium/transient flag
Response	Permeability or permeance; selectivity definition; gas order; replicate count	Diffusivity, solubility, uncertainty, aging/plasticization metrics, mechanical stability	Preserve raw and transformed values; do not infer missing units; flag below-detection results
Provenance	DOI/source; table/figure location; measured/simulated/imputed/equation-derived flag	Extractor, digitization confidence, license, data-processing script and version	Immutable source record; duplicate and conflict audit; documented exclusion rules

Table 2 note: this minimum schema is proposed in the present review rather than reproduced from an external source. FAIR data principles and membrane-specific reporting recommendations informed its design [25,33].

**Table 3:** Representative ML-enabled gas-separation membrane studies classified by decision and strongest reported evidence.

Material	Gas/Separation	Data/Search Scale	ML Decision	Best Validation	Key Quantitative Result	Main Deployment Limitation	Ref.
Polymers	Multiple gases	Sparse experimental permeability matrix	Impute missing gas values	Held-out observed entries	Imputation of missing permeability values (1378 data points per gas) with log-permeability RMSE of 0.07–0.26 (BLR/ERT)	Imputed values are not new measurements; context remains limited	[16]
Polymers	CO_2_/CH_4_ and other pairs	~700 measured; >11,000 screened	Rank exceptional homopolymers	Two selected polymers synthesized and permeation-tested	~700 training polymers; >11,000 homopolymers predicted; 2 synthesized candidates both exceeding the then-relevant CO_2_/CH_4_ upper bound	Realistic mixtures, thin layers and long-term stability not resolved	[17]
Polymers	Six gases	Hundreds measured; >9 million hypothetical	Multitask prediction and discovery	Molecular-dynamics confirmation	>9 million hypothetical polymers screened by multitask graph learning, with molecular-dynamics checks	Polymerization and membrane experiments absent	[18]
Polymers	CO_2_ capture	1169 measured plus generated designs	Generative proposal and screening	Molecular simulation	1169 measured polymers plus generated designs screened by generative design and automated molecular simulation	Synthesis, processing and experimental mixtures absent	[20]
PIM polyimides	Multiple pairs	155,610 candidates; 709 calculations	Physics-informed active screening	Molecular-dynamics validation	155,610 PIM candidates screened with only 709 explicit calculations	No prospective membrane fabrication	[93]
Polymers	O_2_/N_2_, H_2_/CH_4_, H_2_/N_2_	Graph-learning search with prospective candidates	Select explainable high-performance polymers	Selected candidates synthesized and tested	Prospective candidates synthesized and tested, with upper-bound-exceeding performance for several gas pairs	Durability and thin-film evidence remain	[21]
MOFs	H_2_ separation	Large virtual-framework screen	Accelerate ranking	Molecular simulations	CoRE-MOF 2019 membranes screened for six H_2_/X separations (X = CH_4_, N2, H_2_S, O_2_, CO_2_, He) with eight ML methods; top candidates far exceeding the Robeson upper bound	Ideal structures and membrane fabrication gap	[107]
MOFs	Flue-gas CO_2_ capture	3806 MOFs	Rank candidates	Ternary CO_2_/N_2_/H_2_O simulation	3806 MOFs screened; ternary CO_2_/N_2_/H_2_O check performed for leading candidates	No defective polycrystalline membrane test	[108]
MOFs	C_2_H_4_/C_2_H_6_	>10,000 starting frameworks plus generated designs	Inverse framework design	Higher-fidelity transport calculations	>10,000 starting frameworks plus generated designs explored by genetic-algorithm inverse design	Synthesizability and membrane growth unresolved	[19]
COFs and COF/polymer MMMs	He/H_2_	Virtual COF and composite spaces	Screening	Simulation and effective-medium analysis	29,020 COF/polymer and hypoCOF/polymer MMMs computed for six separations; 120 hypoCOFs exceed polymer upper bounds (He/CH_4_, He/N_2_, H_2_/CH_4_, H_2_/N_2_); 18 of 25 polymers carried above the upper bound with COF fillers	Interface and experimental fabrication absent	[113]
MOF/polymer MMMs	CO_2_ capture	Polymer–MOF combinations	Select formulations	Retrospective/computational validation	648 literature MMM data sets (36 MOF types, 41 polymers); test-set R^2^ of 0.77/0.70 for relative permeability/selectivity; guided synthesis exceeded the 2008 CO_2_/CH_4_ upper bound	Limited interface and protocol representation	[125]
MOF/polymer MMMs	CO_2_	54,117 generated combinations	Explainable large-space ranking	Holdout and transfer analyses	54,117 polymer–MOF combinations analyzed with interpretable ensembles	Predominantly equation-derived labels	[128]
CMS membranes	Multiple gases	Literature-derived experimental set	Learn processing-performance relationships	Retrospective ML interpretation	SVR-based regression with R^2^ = 0.932 (RMSE = 0.260, MAE = 0.165) between predicted and experimental CMS permeabilities	Small heterogeneous protocols	[52]
MOF membrane	CO_2_/CH_4_	Simulation + surrogate + process model	Link material to process design	Multiscale computational consistency	ANN surrogate with MSE = 0.0086 and R^2^ = 0.9822, integrated with hollow-fiber process modeling	Single-system simulation; no prospective module test	[36]

**Table 4:** Minimum evidence and reporting checklist for ML-enabled gas-separation membrane studies.

Domain	Minimum Report	Strong Evidence	Red Flag
Research question	Decision, target gas/mixture, membrane architecture and intended use	Pre-specified success criterion linked to process need	“Discover best membrane” without a use condition
Data	Versioned source list; observation schema; units; inclusion/exclusion; label origin	Machine-readable records with provenance, uncertainty and negative outcomes	Untraceable merged spreadsheet or mixed measured/simulated labels
Deduplication	Material and record linkage procedure	Similarity audit and conflict table	Same material/source on both sides of split
Split	Exact rule, random seed and grouping unit	Cluster/family, source, temporal and external tests	Only record-level random split
Preprocessing	Fitted within training folds; missing-data procedure	End-to-end pipeline released	Imputation/scaling before split
Baselines	Mean/linear, descriptor + tree ensemble and relevant physical model	Statistical comparison across repeated grouped splits	Complex model compared only with a weak baseline
Metrics	MAE/RMSE in physical or log units; rank metric if screening; calibration/coverage	Metrics by material family and operating domain	R^2^ alone or undefined averaging across gases
Applicability	Training-domain coverage and OOD indicator	Calibrated intervals on external chemistry	Point predictions without domain warning
Interpretability	Feature provenance and stability across resamples	Valid chemical counterfactuals or controlled intervention	Causal language from one attribution plot
Candidate selection	Search space, constraints, acquisition/ranking rule and candidate denominator	Time-stamped protocol including failed candidates	Post hoc cherry-picking of successful candidates
Experimental test	Full fabrication and gas-test conditions; replicates and uncertainty	Thin-film, mixed-gas, aging, humidity/contaminants as relevant	Pure-gas bulk-film value used as deployment proof
Reproducible package	Code, trained model, environment and data license/version	Executable workflow and external reproduction	DOI list without extracted data or model settings

Table 4 note: this checklist is proposed in the present review and is not reproduced from a single guideline. Its data and provenance elements synthesize FAIR principles [33], its split and redundancy elements follow leakage-control evidence [29], and its uncertainty and interpretation elements reflect current benchmarking and explainability practice [34,96]. A separate reference column is therefore unnecessary because the rows form one integrated normative framework rather than summaries of distinct primary studies.

**Table 5:** Research priorities and measurable milestones for decision-grade ML in gas-separation membranes.

Horizon	Priority Action	Why It Matters	Measurable Milestone
0–2 years	Adopt a contextual observation schema and persistent material/specimen IDs	Removes label ambiguity and enables deduplication	Public datasets report the Table 2 fields and measured/simulated/imputed flags
0–2 years	Establish gas-pair benchmark suites with chemistry-, source- and time-disjoint splits	Separates interpolation from discovery generalization	Leaderboards publish random and OOD metrics with identical training data
0–2 years	Require calibrated UQ and simple/physical baselines	Prevents overconfident ranking and unjustified model complexity	External-set interval coverage and applicability domain reported by default
1–3 years	Publish negative fabrication, stability and below-detection outcomes	Enables feasibility learning and reduces publication bias	Repositories accept structured failure taxonomies and censored values
1–4 years	Build prospective multi-candidate challenges	Tests selection policies rather than retrospective fit	Candidate rules time-stamped; all attempts and costs reported
2–5 years	Create mixed-gas thin-film datasets with aging, humidity and contaminants	Closes the dominant proxy gap	Cross-laboratory reference membranes and standardized protocols available
2–5 years	Develop hierarchical and multi-fidelity models for chemistry–process–morphology	Connects controllable fabrication to transport	Prospective formulation models outperform chemistry-only baselines
3–6 years	Couple material/UQ models to module and process optimization	Aligns discovery with energy, purity, recovery and lifetime	Candidate rankings shown to change with process conditions and uncertainty
4–8 years	Federated closed-loop membrane laboratories	Shares learning while respecting proprietary data and local equipment	Multiple laboratories reproduce model-selected gains using a common protocol

## Data Availability

No new data were created or analyzed in this review.

## Abbreviations

AL, active learning; CMS, carbon molecular sieve; COF, covalent organic framework; GNN, graph neural network; ML, machine learning; MMM, mixed-matrix membrane; MOF, metal–organic framework; OOD, out of distribution; PIM, polymer of intrinsic microporosity; UQ, uncertainty quantification.
